# When Precision Meets Penmanship: ChatGPT and Surgery Documentation

**DOI:** 10.7759/cureus.40546

**Published:** 2023-06-17

**Authors:** Alexander Robinson, Shaurya Aggarwal

**Affiliations:** 1 General Surgery, Mid and South Essex NHS (National Health Service) Foundation Trust, Chelmsford, GBR

**Keywords:** laparoscopic surgery, appendicectomy, nhs, chatgpt, surgical documentation

## Abstract

ChatGPT (Chatbot Generative Pre-Trained Transformer) is an artificial intelligence with several potential applications in the field of medicine. As a large language model, it is particularly good at generating text. This study investigates the use of ChatGPT in constructing operation notes for laparoscopic appendicectomy, one of the most common surgical procedures in the UK. We prompted ChatGPT-4, the latest generation of ChatGPT, to produce operation notes for laparoscopic appendicectomy, which were then evaluated against ‘Getting It Right First Time’ (GIRFT) recommendations. GIRFT is an organisation that has collaborated with the National Health Service (NHS) to improve surgical documentation guidelines. Excluding certain items documented elsewhere in patient records, the generated notes were assessed against 30 key points in GIRFT recommendations. This process was repeated three times to obtain an average score. Our results showed that ChatGPT generated operation notes in seconds, with an average coverage of 78.8% (23.66 out of 30 points) of the GIRFT guidelines, surpassing average compliance with similar guidelines from the Royal College of Surgeons (RCS). However, the quality of ChatGPT’s output was found to be dependent on the quality of the prompt, highlighting the need for verification of the generated content. Additionally, secure integration with electronic health records is required before ChatGPT can be adopted into the NHS.

## Introduction

ChatGPT (Chatbot Generative Pre-Trained Transformer) is an artificial intelligence developed by Open AI. It has several potential applications in the field of medicine, many of which have been published elsewhere. As a large-scale language model, it is particularly useful in generating text. In this study, we investigate the use of ChatGPT in constructing an operation note.

Writing an operation note is a crucial part of any surgical procedure. It allows future medical professionals to know what happened at the time of surgery. An accurate and detailed description of these events ensures patient safety and continuity of care. Furthermore, there are important medicolegal implications in providing clear documentation to prevent future litigation claims that often exploit gaps and ambiguities in patient notes. In the UK, the current cost of litigation against the National Health Service (NHS) is a staggering £128.6 billion [1] or approximately 1/3 of the yearly budget. Based on the previous litigation claims and feedback from NHS panel firm lawyers, the organisation ‘Getting it right first time’ (GIRFT) has produced guidelines on documentation of operation notes [2]. The guidelines currently cover five common surgical procedures: appendicectomy, cholecystectomy, inguinal hernia repair, laparotomy, and bowel resection.

In a busy operating list, where efficiency is key, ChatGPT has the potential to help surgeons streamline the process of writing an operation note whilst making sure it is thorough and accurate. By reducing the time spent on documentation, it could alleviate some of the administrative burdens on surgeons and allow them to have more rest between cases. The significance of addressing surgeon fatigue and the importance of adequate breaks to prevent burnout is becoming increasingly recognised [3]. A study published in the Lancet Digital Health has demonstrated that ChatGPT can generate adequate discharge summaries from a short prompt [4]. This study investigates whether ChatGPT can do the same for operation notes, whilst maintaining GIRFT standards. The focus of this investigation is on laparoscopic appendicectomy, one of the most common surgical procedures performed in the UK.

## Technical report

We asked ChatGPT-4, the latest generation of GPT to produce an operation note for a laparoscopic appendicectomy based on the following prompt:

"Complete an operation note for 'A 25 yo male, laparoscopic appendicectomy for migratory RIF pain and tenderness. GA. 40ml 0.5%. 3 port. Appendix inflamed perforated, nil else healthy base. Suction washout. Endoloops and diathermy used. Closure. 5 days co-amox. No drain."

The results were then evaluated against the GIRFT recommendations on best practice for laparoscopic appendicectomy documentation [5]. Although the guidelines encompass 34 criteria, they acknowledge that certain elements might be documented elsewhere in the patient’s record, such as accident and emergency (A&E) assessment, ward round entries, and a separate WHO (World Health Organisation) surgical checklist. Consequently, in the context of evaluating the comprehensiveness of an operative note, we excluded 'time of decision to operate,' 'consent process,' 'WHO checklist’, and the surgeon's signature, considering the latter's incompatibility with digitisation. We assessed ChatGPT’s response against the remaining 30 points, a score of 1 was given if the recommendation was documented, and a score of 0 was given if it was not. We then calculated a total score out of 30. This process was repeated three times with the same prompt to obtain an average score (Table 1). These generated outputs and results are as follows.

**Table 1:** ChatGPT’s response as compared to GIRFT recommendations for documentation of practice in all patients undergoing laparoscopic appendicectomy. GIRFT: Getting It Right First Time, VTE: venous thromboembolism.

GIRFT recommendations	Score for operation note 1	Score for operation note 2	Score for operation note 3
Indications for operation	1	1	1
Names + grades of surgeons	1	1	1
Names + grades of anaesthetists	0	1	1
Date and time of procedure	0	0	1
Drugs given before or during surgery	1	1	1
Insertion of urinary catheter	0	0	0
Patient position and skin preparation	1	1	1
Location of incisions	1	1	1
Technique to enter peritoneal cavity	1	1	1
Confirm laparoscope was inserted into the abdomen under direct vision	1	1	1
Position and size of other ports inserted	1	1	1
Level of intra-abdominal pressure	1	1	1
Record patient was placed in Trendelenburg position with left side down	0	0	0
Findings of surgery	1	1	1
Record if the appendix appeared normal	1	1	1
Location of appendix	0	0	0
Record if the appendix was stapled or ligated between endo-loops	1	1	1
Record if the appendiceal artery was divided with cautery or between clips	1	1	1
Record if the remainder of mesoappendix was dissected and if base of caecum was healthy	1	1	1
Volume of irrigation used	1	1	1
Endoscope bag used for extraction of appendix	1	1	1
Record if appendix removed whole or any residual left behind	1	1	1
State that tissue sample was sent to pathology	0	0	1
Record haemostasis was achieved	1	1	1
Record ports were removed under direct vision	1	1	1
Record any intra-operative complications	1	1	0
Details of closure and use of drains	1	1	1
Estimate of anticipated blood loss	1	0	1
Post-operative planning including antibiotics, blood tests, location of transfer, removal of drains, VTE thromboprophylaxis, discharge plans, follow-up	1	1	1
Record any images taken during procedure	0	0	0
	Total score: 23/30	Total score: 23/30	Total score: 25/30

ChatGPT generated operation note 1

Procedure: Laparoscopic Appendicectomy

Patient: 25-year-old male

Indication: Migratory right iliac fossa pain and tenderness

Anaesthesia: General anaesthesia

Surgeon: [Surgeon's Name]

Assistant: [Assistant's Name]

Scrub Nurse: [Scrub Nurse's Name]

Circulating Nurse: [Circulating Nurse's Name]

Preoperative Diagnosis: Acute appendicitis

Postoperative Diagnosis: Acute perforated appendicitis

Findings: Inflamed and perforated appendix, healthy base, no other abnormalities

Procedure Details:

1. The patient was brought to the operating room and placed in the supine position. General anaesthesia was induced, and the patient was intubated without complications.

2. The abdomen was prepped and draped in a sterile fashion.

3. A 40 ml solution of 0.5% bupivacaine was infiltrated at the planned incision sites for local anaesthesia.

4. A 3-port technique was utilized for the laparoscopic appendicectomy. A 10 mm umbilical port was inserted using the Hasson technique, and the abdomen was insufflated with CO2 to a pressure of 15 mmHg. Two additional 5 mm ports were placed in the left lower quadrant and suprapubic region under direct visualization.

5. Upon inspection, the appendix appeared inflamed and perforated. No other abnormalities were identified.

6. The mesoappendix was divided using a combination of endoloops and diathermy.

7. The base of the appendix was secured with two endoloops, and the appendix was divided between the loops using diathermy.

8. The specimen was retrieved using an endoscopic retrieval bag and removed through the umbilical port.

9. Copious suction and washout of the peritoneal cavity were performed.

10. Haemostasis was confirmed, and the appendiceal stump was inspected for any signs of bleeding or contamination.

11. The ports were removed under direct visualization, and the fascia at the umbilical port site was closed using absorbable sutures.

12. The skin incisions were closed with subcuticular absorbable sutures and covered with sterile dressings.

13. The patient was extubated and transferred to the recovery room in stable condition.

Estimated Blood Loss: Minimal

Complications: None

Disposition: The patient was admitted for postoperative care and monitoring.

Postoperative Instructions: The patient will receive a 5-day course of co-amoxiclav as per hospital protocol. No drain was placed.

Follow-up: The patient is to follow up with the surgeon in 2 weeks for a postoperative check-up and suture removal, or earlier if any complications arise.

ChatGPT generated operation note 2

Patient: 25-year-old male

Procedure: Laparoscopic Appendectomy

Indication: Migratory right iliac fossa (RIF) pain and tenderness

Anaesthesia: General Anaesthesia (GA)

Local Anaesthesia: 40 ml of 0.5% Bupivacaine

Surgeon: [Surgeon's Name]

Assistant: [Assistant's Name]

Anaesthetist: [Anaesthetist’s Name]

Scrub Nurse: [Scrub Nurse's Name]

Circulating Nurse: [Circulating Nurse's Name]

Description of Procedure:

1. The patient was brought to the operating room and placed in a supine position on the operating table. General anaesthesia was induced by the anaesthesia team, and the patient was intubated without any complications.

2. The abdomen was prepped and draped in a sterile fashion. A time-out was performed to confirm the correct patient, procedure, and operative site.

3. A 1 cm infraumbilical incision was made, and a Veress needle was inserted into the peritoneal cavity. The abdomen was insufflated with CO2 to a pressure of 15 mmHg. A 10 mm trocar was introduced through the infraumbilical incision.

4. Under direct vision, two additional ports were inserted: a 5 mm port in the left lower quadrant and another 5 mm port in the suprapubic area.

5. A laparoscopic exploration of the abdominal cavity was performed. The appendix was found to be inflamed and perforated. No other abnormalities were detected.

6. The mesoappendix was divided using diathermy. Two endoloops were applied to the base of the appendix, and the appendix was transected between the endoloops.

7. The specimen was placed in an endoscopic retrieval bag and removed through the 10 mm infraumbilical port.

8. The abdominal cavity was thoroughly irrigated with warm saline, and suction was used to remove any residual fluid.

9. The three trocar sites were inspected for bleeding or injury. Haemostasis was confirmed.

10. The pneumoperitoneum was deflated, and the trocars were removed under direct vision.

11. The fascia of the 10 mm infraumbilical port site was closed using an absorbable suture. All incisions were closed with absorbable subcuticular sutures and covered with sterile adhesive dressings.

12. The patient was extubated and transferred to the recovery room in stable condition.

Postoperative Plan:

1. The patient was prescribed a 5-day course of co-amoxiclav as antibiotic prophylaxis.

2. Pain management with appropriate analgesics.

3. No drain was placed.

4. Monitor for signs of infection or complications.

5. Encourage early ambulation and a gradual return to normal activities.

6. Schedule a follow-up appointment in 1-2 weeks for wound assessment and suture removal, if needed.

The patient tolerated the procedure well, and there were no intraoperative complications.

ChatGPT generated operation note 3

Procedure: Laparoscopic Appendicectomy

Date: [Procedure Date]

Patient: 25-year-old male

Indications: Migratory right iliac fossa (RIF) pain and tenderness

Anaesthesia: General anaesthesia (GA)

Surgeon: [Surgeon's Name]

Assistant: [Assistant's Name]

Anaesthesiologist: [Anaesthesiologist’s Name]

Nurse: [Nurse's Name]

Findings: Inflamed, perforated appendix; no other abnormalities identified

Procedure Details:

1. The patient was brought into the operating room and placed in a supine position with arms tucked.

2. General anaesthesia was administered, and the patient was intubated without complications.

3. The abdomen was prepared and draped in a sterile fashion.

4. A 10-mm umbilical incision was made for the camera port, and pneumoperitoneum was established with a Veress needle. Intra-abdominal pressure was maintained at 12 mmHg.

5. Two additional 5-mm trocars were inserted under direct vision in the left lower quadrant and suprapubic region, creating a 3-port configuration.

6. The laparoscope was introduced, and a thorough examination of the abdominal cavity was performed, revealing an inflamed, perforated appendix with no other abnormalities.

7. The mesoappendix was carefully dissected using diathermy, and the appendiceal artery was controlled with endoloops.

8. The base of the appendix was doubly ligated with endoloops, ensuring a healthy margin, and then transected using diathermy.

9. The specimen was placed in an endoscopic retrieval bag and removed through the umbilical port.

10. The peritoneal cavity was copiously irrigated with warm saline, and suction was applied for washout to remove any residual infectious material.

11. Haemostasis was confirmed, and no drain was placed.

12. All trocars were removed under direct vision, and the fascia at the umbilical port site was closed with absorbable sutures.

13. The skin incisions were closed with subcuticular sutures and dressed with sterile adhesive strips.

14. The patient was extubated and transferred to the recovery room in stable condition.

Estimated Blood Loss: Minimal

Specimen: Appendix

Postoperative Diagnosis: Acute perforated appendicitis

Postoperative Care:

1. The patient was prescribed a 5-day course of co-amoxiclav (co-amox) for postoperative antibiotic coverage.

2. Pain management, as needed, using a multimodal analgesic regimen.

3. Early ambulation and a gradual return to normal activities as tolerated.

4. Wound care and monitoring for signs of infection.

5. Follow-up appointment in 2 weeks to assess recovery and wound healing.

6. The patient was advised to seek medical attention promptly if experiencing fever, worsening abdominal pain, or any signs of infection.

## Discussion

Whilst the style and language of an operation note is often a personal preference of the surgeon, the recommended information to be included is largely standardised and/or repeated. Many surgeons keep templates on their personal devices and change the details of the current case. Templates have been shown to improve compliance with Royal College of Surgeons (RCS) guidelines [6,7]. In this context, our proof-of-concept experimentation with ChatGPT is promising. ChatGPT was able to quickly create an operation note that was clear and succinct. It was able to document 78.8% of the operation details as recommended by GIRFT guidelines. Previous studies comparing laparoscopic appendicectomy notes to similar guidelines produced by the RCS show an average compliance of 56.1%-66% [6,7]. This is particularly useful for operations for which the surgeon does not already have a prepared template.

Notable omissions in all three of ChatGPT’s operation notes were urinary catheter insertion, patient positioning in Trendelenburg position, location of the appendix, and record of any images taken. Upon writing an updated prompt to incorporate these details, ChatGPT was able to quickly produce an amended operation note and remembered to include these details in subsequent operation notes. It should be noted that while ChatGPT can record ‘conversations’ within the same interaction, it does not have the capability to retain information across different sessions. It is also important to note that the output quality appeared to be related to the quality of the prompt in our early experimentation. When the prompt lacked specific information, such as the consent process or the type of local anaesthetic used, ChatGPT occasionally failed to document these details. Consequently, its ‘score’ marked against the GIRFT recommendations varied with every output. Despite consistently generating detailed operation notes, this highlights the necessity for verification of the generated content.

It appeared that ChatGPT was best used as an intelligent ‘assistant’. As there are many ways an operation can be performed, the ‘assistant’ first has to get to know how a surgeon performs an operation. For instance, one operation note reported a Veress needle insufflation which is not the common technique of entry into the abdomen in the UK; the Hasson technique is more common. However, once told, ChatGPT will include the Hasson technique in subsequent notes.

While ChatGPT provides insight into how large language models can help alleviate administrative burdens, it is not yet ready for widespread adoption throughout the NHS. It is important to be cautious of its potential drawbacks. Its use in writing operation notes may not be applicable to all types of surgeries, especially less common or more specialised procedures. These may not have been represented in the data that the model was trained on. Additionally, given that its knowledge is currently only up to 2021, it will not be aware of surgical techniques that have been adopted beyond this timeframe. Additionally, there is a risk of becoming over-reliant on the automated process without adequate checking of the produced document.

Another issue is that confidential patient information should not be stored on insecure servers abroad. If intelligent large language models can be integrated into secure electronic health records, patient details such as past medical history, operation dates, surgeon, and anaesthetist information could be auto-filled making the process even more streamlined. NHS trusts are currently trialling the use of AI-powered voice recognition with various programmes such as dictate IT [8]. Open AI offers the Application Programming Interface (API) of ChatGPT to third-party developers to be integrated into their own products which may allow more widespread licencing and use within the NHS. These technologies could be integrated with a large language model (LLM) such as ChatGPT to allow secure voice dictation of comprehensive operation notes and could allow the outputs to be seamlessly processed into operative notes instead of requiring the user to copy and paste into a Word document to print as in the current iteration. Furthermore, these operation notes could be integrated with medical inpatient notes to streamline the creation of discharge summaries.

## Conclusions

ChatGPT can serve as an assistant for writing detailed operation notes, helping to save time and alleviating pressure on medical professionals, a factor shown to increase errors. However, care must be taken to ensure that the generated note contains the correct procedure technique and case-specific details. After repeated uses and requests, ChatGPT does become an intelligent and accurate surgical note-making assistant.
